# Nicotinic acid modulates intracellular calcium concentration and disassembles the cytoskeleton

**DOI:** 10.3892/mmr.2014.2576

**Published:** 2014-09-18

**Authors:** JIEJING LI, YANXI LI, PENGHUI ZHANG, HUA NIU, YU SHI

**Affiliations:** 1 Department of Clinical Laboratory, Children’s Hospital of Chongqing Medical University, Chongqing 400014, P.R. China; 2 Laboratory of Developmental Diseases in Childhood of Education Ministry, Key Laboratory of Pediatrics in Chongqing, Chongqing International Science and Technology Cooperation Center for Child Development and Disorder, Children’s Hospital of Chongqing Medical University, Chongqing 400014, P.R. China; 3 Clinical Laboratory Centre, The First People’s Hospital of Yunnan Province, Kunming, Yunnan 650032, P.R. China

**Keywords:** nicotinic acid, calcium, cytoskeleton, xenopus

## Abstract

Nicotinic acid (NA), a member of the vitamin B family, is well known for its functions in the treatment and prevention of atherosclerosis due to decreasing plasma levels of low-density lipoprotein cholesterol. In recent years, the major side effect of NA, cutaneous flushing, has also attracted extensive attention. However, the effects of NA in other aspects of physiology or cell biology have remained elusive. The present study provided evidence that high concentrations of NA were able to first reduce and later elevate intracellular [Ca^2+^] in the NIH3T3 cell line. The reduction of the intracellular Ca^2+^ concentration was achieved within the initial 10 sec, and was preceded by a gradual elevation of intracellular [Ca^2+^]. Notably, marked accumulation of opaque materials in the perinuclear region was observed in NIH3T3 cells treated with 70 mM NA. Further analysis revealed that treatment with 70 mM NA for 1 h disassembled the microtubule and F-actin cytoskeleton systems and resulted in β-tubulin degradation in an ubiquitin-proteasome-dependent manner. These data indicated that high concentrations of NA disrupted cytoskeleton structures, which may have contributed to minus end (nucleus region) to plus end (cell membrane region)-directed transport processes and resulted in the deposition of material in the perinuclear region. Artificially increasing [Ca^2+^] adding CaCl_2_ to the culture media effected the disassembly of F-actin, while it had no apparent effect on microtubules. These results suggested that the disruption of the cytoskeleton systems was not entirely due to the NA-induced elevation of [Ca^2+^]. Finally, microinjection of NA into xenopus embryos blocked the transport of melanosomes to the peripheral cellular area. In conclusion, the present study indicated that NA disassembles F-actin and microtubule systems, thereby blocking cytoskeleton-dependent intracellular transport.

## Introduction

The milestone results reported by Altschul *et al* (1) >50 years ago demonstrated that nicotinic acid (NA) has the capacity to decrease plasma lipids. As a result, this water soluble vitamin B family member has been widely used clinically for the treatment and prevention of atherosclerosis and other lipid-metabolic disorders (2,3). At present, NA is one of the most effective agents that offers protection against cardiovascular risk factors by increasing high density lipoprotein (HDL) levels, while simultaneously decreasing very low density lipoprotein (VLDL) and low density lipoprotein (LDL) levels (4). The major side effect of NA is cutaneous vasodilatation, also known as ‘flush’, which limits its clinical utility and applications (5). NA functions by downregulating intracellular cyclic adenosine monophosphate (cAMP), the major intracellular mediator of prolipolytic stimuli, and subsequently decreases cellular levels of free fatty acids (5). Notably, prostaglandin has been demonstrated to have a vital role in flushing (6,7). Anti-lipid and flush effects are mediated by its G protein-coupled receptor GPR109A (8,9). Despite extensive studies in the field of lipid metabolism, the effects of NA on other aspects of cellular physiology remain elusive. Previously, several groups have demonstrated that NA elevates intracellular [Ca^2+^] in neutrophil (10), macrophage (8) and CHOK1 cell lines (9) in a GPR109A-dependent manner. Elevation of intracellular [Ca^2+^] may transduce a number of different signaling pathways in different cell types. In the present study, variations in intracellular Ca^2+^ levels were observed under incubation with different concentrations of NA, and long-term (1 h) effects on the NIH3T3 cell line and its cytoskeleton were analyzed.

## Materials and methods

### Cell culture

CHO-K1 cells (cat.no CCL-61; American Type Culture Collection; Manassas, VA, USA) were grown in F12 medium (11765047; Life Technologies, Grand Island, NY, USA) supplemented with 10% fetal calf serum (FCS; 16170086; Life Technologies). The 293T cells (CRL-3216; American Type Culture Collection) were grown in Dulbecco’s modified Eagle medium (DMEM; 12430047; Life Technologies) with 10% fetal calf serum (FCS). The NIH3T3 cells (CRL-1658; American Type Culture Collection) were cultured in DMEM with 10% FCS.

### Time lapse measurement of intracellular [Ca^2+^]

The cells (2×10^4^/well) were allowed to adhere to a sterile 96-well cell culture plate (Greiner Bio-One) and incubated with Fluo3 acetoxymethyl (AM) Ca^2+^ indicator (Molecular Probes, Invitrogen Life Technologies, Carlsbad, CA, USA) for 1 h at 37°C. The Ca^2+^ levels were assessed by measuring the fluorescent intensity using a Zeiss LSM 510 META confocal microscope and Zeiss Lsm Image Examiner software (FV10-ASW 2.1 Viewer; Carl Zeiss, Jena, Germany) was applied for quantitative analysis.

### Fluorescent immunohistochemistry

The cells were fixed for 10 min with 3.7% paraformaldehyde (Sigma, St. Louis, MO, USA) and permeabilized with 0.2% Triton X-100 (Sigma). The F-actin stress fibers were labeled with Texas Red-X phalloidin (Molecular Probes, Invitrogen Life Technologies). The microtubule filaments were stained with monoclonal mouse anti-β-tubulin antibody (1:200; E1C-601; EnoGene, New York, NY, USA) and the secondary antibody was goat-anti-mouse-fluorescein isothiocyanate (1:200; Sigma).

### Western blot analysis

The cells were incubated with MG132 (10 μM; Santa Cruz Biotechnology, Inc., Santa Cruz, CA, USA) and/or NA and collected at the appropriate time. The cells were boiled at 100°C in Lämmli buffer for 5 min. The following antibodies were used: Mouse anti-β-tubulin monoclonal antibody (1:10,000; EnoGene E1C-601; EnoGene); mouse anti-β-actin monoclonal antibody (1:10,000; ab6276; Abcam, Massachusetts, MA, USA); rabbit anti-H3 polyclonal antibody (1:10,000; H0164; Sigma); horseradish peroxidase (HRP)-goat anti mouse antibody (1:10,000; A3673; Sigma); HRP-goat anti-rabbit antibody (1:10,000; sc-2030; Santa Cruz Biotechnology, Inc.).

### Xenopus embryo manipulation and microinjection

*In vitro* embryo fertilization and culture were conducted as described previously (11). For each embryo, 70 ng of NA was injected at the 2 cell stage. The microinjection procedure was performed as described previously (12). The use of Xenopus embyos in the study was approved by the Ethics Comittee of the Children’s Hospital of Chongqing Medical University (Chongqing, China).

## Results

### NA regulates intracellular Ca^2+^ mobilization

**Figure 1:**
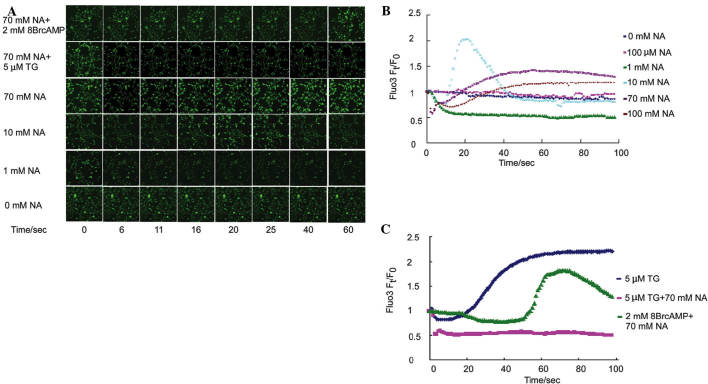
Time lapse assessment of intracellular [Ca^2+^] levels upon exposure to different concentrations of NA. (A) Visualization of intracellular [Ca^2+^] upon exposure to different concentration of nicotinic acid and other drugs by staining with Fluo3 acetoxymethyl Ca^2+^ indicator; (B) time lapse statistical curve of intracellular Ca^2+^ levels upon exposure to different NA concentrations; (C) TG inhibits the elevation of [Ca^2+^] induced by NA (pink curve) and cAMP analog 8-Br-cAMP delays and alleviates NA-induced primary [Ca^2+^] decrease (yellow curve). F_0_: Fluo3-labeled intensity prior to exposure to NA or other drugs; F_t_: Fluo3-labeled intensity at time-point t. NA, nicotinic acid; TG, thapsigargin.

To examine the time lapse effect of NA on intracellular free Ca^2+^ mobilization, Fluo3-labeled NIH3T3 cells were incubated with different concentrations of NA, and the fluorescence intensity was simultaneously assessed over 100 sec. The fluorescence intensity reflected the intracellular free Ca^2+^ concentration. Previous studies have demonstrated that 100 μM NA induced transient intracellular [Ca^2+^] elevation in CHO-K1 cells (9), macrophages (8) and matured neutrophils (10) within one to several minutes. In the NIH3T3 cell line, 100 μM NA did not alter the intracellular Ca^2+^ mobilization (Fig. 1B, pink curve). The NIH3T3 cells were further exposed to a wider span of NA concentration gradients and the Ca^2+^ mobilization was assessed. At a 1 mM NA, the intracellular Ca^2+^ levels decreased by 50% within 10 sec and no elevation was detected during the entire process (Fig. 1A and B, green curve). In the 10 mM NA exposure group, intracellular free Ca^2+^ reduced precipitously similarly to the observations at 1 mM NA; however, a transient sharp elevation-reduction n-turn like curve of Ca^2+^ mobilization was observed (Fig. 1A and B, light blue curve). Consistent with the 10 mM group, both 70 mM (Fig. 1B, purple curve) and 100 mM (Fig. 1B, brown curve) NA decreased intracellular free [Ca^2+^] within the first several seconds, and secondarily, triggered an elevation in intracellular free [Ca^2+^]. Of note, secondary increase in [Ca^2+^] was slower with increasing NA concentration. In addition, NA-induced secondary [Ca^2+^] was inhibited by thapsigargin (TG; Fig. 1A and C, pink curve), an endoplasmic reticulum (ER) Ca^2+^-ATPase pump inhibitor, which induces Ca^2+^ release from the ER. Furthermore, the NA-induced decrease in primary intracellular [Ca^2+^] was delayed by the addition of 2 mM of the cAMP analog 8Br-cAMP (Fig. 1A and C, green curve). These data suggested that the reduction of cAMP levels by NA may be responsible for the primary transient ER decrease and Ca^2+^ release by the endoplasmic reticulum (ER) contributed to the later observed Ca^2+^ elevation.

### NA disassembles the cytoskeleton and deposits opaque materials at the perinuclear region

**Figure 2:**
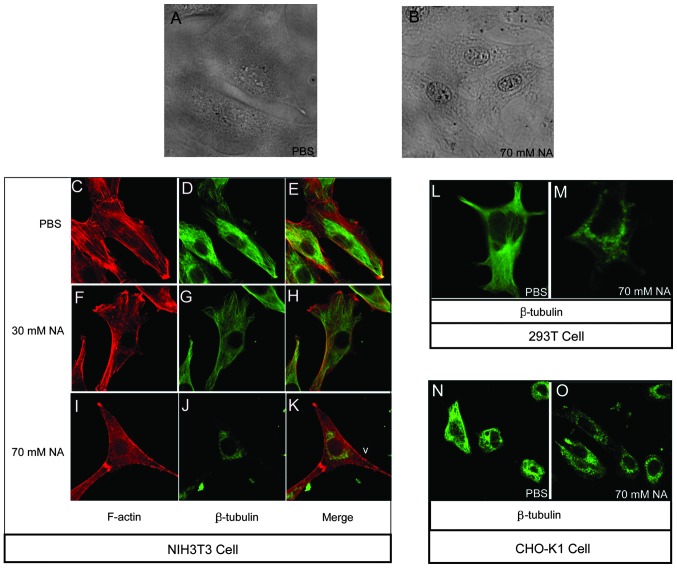
NA disassembles the cytoskeleton and deposits opaque materials at the peri-nuclear region. Compared with (A) the PBS-incubated control group, (B) 70 mM NA results in evident nucleolar accumulation of opaque materials at peri-nucleus regions in NIH3T3 cells. In the PBS-incubated NIH3T3 cells, (C) F-actin and (D) microtubules are filament-rich as is apparent in (E) the merged image. Exposure to 30 mM NA for 1 h partially disassembled (F) F-actin and (G) microtubules as is apparent in (H) the merged image. 70 mM NA completely disassembled (I) F-actin and (J) microtubules as apparent in (K) the merged image. Microtubule structure was disassembled when exposed to 70 mM NA for 1 h in both (L and M) 293T cells and (N and O) CHO-K1 cells. (Magnification, ×250). NA, nicotinic acid.

Besides intracellular Ca^2+^ wave variation, the results revealed that an accumulation of unidentified opaque material at the perinuclear region, forming a ring-type structure, as well as at the nucleolus, was markedly evident in the NIH3T3 cells following incubation with 70 mM NA (Fig. 2B). However, in the phosphate-buffered saline (PBS)-treated control group, the NIH3T3 cells exhibited a spread morphology and no perinuclear ring or dim nucleolus phenotypes (Fig. 2A). The highly visible nucleolus in the NA-treated cells suggested the activation of synthetic processes; however, the manner in which the perinuclear opaque rings formed remains elusive. Cytoskeletal organization has a number of important roles in intracellular transport processes. The assembly-disassembly homeostasis of F-actin and microtubules are regulated by numerous factors, including variations in intracellular [Ca^2+^] (13–17). The NA-induced phenotypes identified in the present study allowed for the following hypothesis: NA changes intracellular [Ca^2+^], thereby obstructing cytoskeletal integrated organization, then affecting cytoskeleton-dependent intracellular transport and finally causing the accumulation of material at the perinuclear area, forming an opaque ring like structure. To confirm this hypothesis, F-actin and microtubule structures were observed with Texas Red-X phalloidin and anti-β-tubulin antibodies, respectively, under different concentrations of NA. In the PBS-treated control group, the F-actin (Fig. 2C) and microtubules (Fig. 2D) were normally patterened, as shown in the merged image in Fig. 2E. The F-actin (Fig. 2F) filaments began to disassemble, forming punctuated spots and the microtubules (Fig. 2G) exhibited weaker staining following treatment with 30 mM/1 h NA. Following incubation with 70 mM/1 h NA, the F-actin (Fig. 2I) and microtubule (Fig. 2J) cytoskeletons (merged in Fig. 2K) were completely disassembled, and the liberated microtubule residues accumulated at the distal end of the filopodia and perinuclear region (Fig. 2J). Further analysis confirmed the occurrence of microtubule disassembly with 70 mM NA in the 293T (Fig. 2L and M) and CHO-K1 cell lines (Fig. 2N and O). These data indicated that NA dissociates the F-actin and microtubule cytoskeleton, which may affect intracellular transport in a dose-dependent manner.

### Abnormal increases in the Ca^2+^ concentration contribute to the disassembly of F-actin

**Figure 3:**
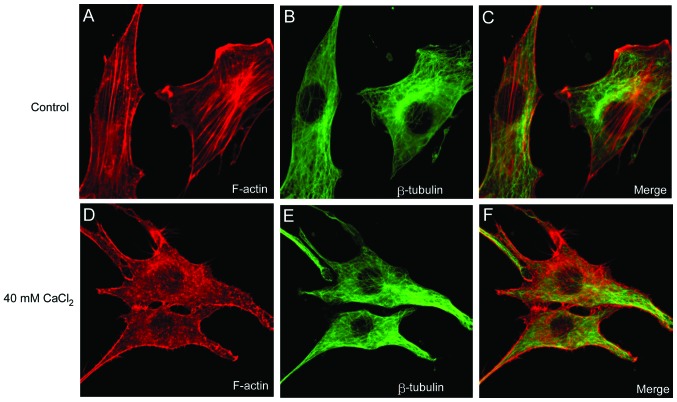
External addition of CaCl_2_ to the culture media and 1 h incubation disrupted the F-actin filaments, stained with Texas Red-X phalloidin, but did not affect the microtubules, stained with fluorescein isothiocyanate-conjugated antibody. (Magnification, ×250).

To further elucidate the association between changes in [Ca^2+^] and the disassembly of the cytoskeleton, a CaCl_2_ solution was added directly into the NIH3T3 cell culture media and the cytoskeleton structure was examined following 1 h of incubation. Consistent with the effect of NA demonstrated above, 40 mM CaCl_2_ disrupted F-actin filaments (Fig. 3A) into punctuate G-actin spots (Fig. 3D). However, artificial increases in [Ca^2+^] did not affect the microtubular structure (Fig. 3E) compared with that of the control group (Fig. 3B). The results implied that the Ca^2+^ wave induced by NA may be involved in the disruption of F-actin filaments, but not in the disassembly of the microtubular polymer structure.

### Depolymerized microtubule subunits undergo ubiquitin-proteasome degradation

**Figure 4:**
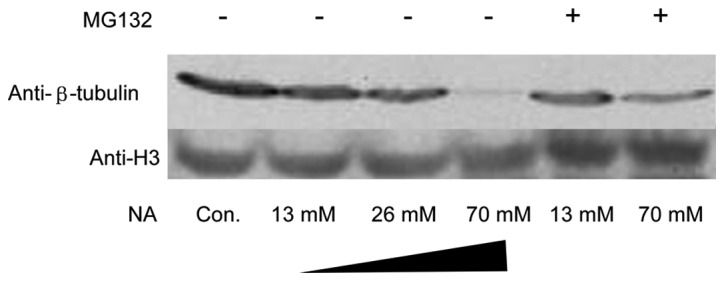
NA caused microtubule component β-tubulin degradation in an ubiquitin-proteasome pathway. NA, nicotinic acid.

Microtubules consist of α-tubulin and β-tubulin hetero-subunits. The microtubules were labeled with anti-β tubulin antibody. Under exposure to 70 mM NA for 1 h, not only did the microtubule-stained pattern change, but also its immunofluorescent intensity decreased significantly (Fig. 2J, M and O). To confirm these results, a total amount of β tubulin was analyzed using western blot analysis. As expected, NA markedly downregulated β-tubulin at the protein level. In addition, MG132, an inhibitor of the ubiquitin-proteasome pathway, was able to reverse β-tubulin reduction (Fig. 4). However, there were no significant changes in F-actin monomer protein G-actin (data not shown).

### NA blocks melanosome intracellular transport in xenopus embryos

**Figure 5:**
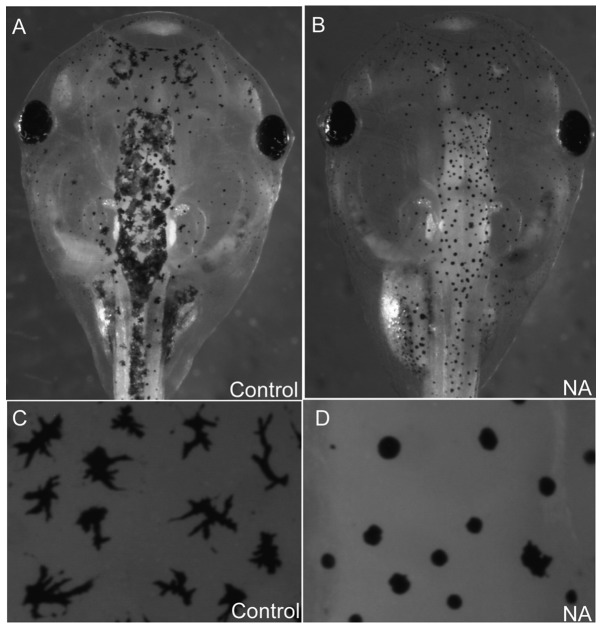
Microinjection of 70 ng NA into xenopus embryos inhibited the melanosome transport. NA, nicotinic acid. (Magnification, ×30).

In the cultured cells NA disrupted cytoskeletal integrity and may have inhibited intracellular trafficking. To investigate the effect of NA on intracellular transport processes *in vivo*, 70 ng NA was microinjected into xenopus embryos and its effect on melanosome transport in melanocytes was observed. Melanosomes either disperse or aggregate along microtubules and F actin-filaments (18). As they are easy to observe, melanocytes represent a reliable system for investigating intracellular transport. In normal embryos, the melanosomes disperse uniformly in a dendritic type manner (Fig. 5A and C). By contrast, in NA-microinjected embryos, melanosome transport was blocked and exhibited an aggregated disc type morphology (Fig. 5B and D), suggesting that NA blocked intracellular transport processes *in vivo*.

## Discussion

Previous studies have demonstrated that 100 μM NA evokes intracellular [Ca^2+^] within several minutes (8–10); however, the detailed mechanisms underlying this effect remains elusive. In the present study, intracellular [Ca^2+^] was assessed in a time lapse manner upon exposure to NA. NIH3T3 cells required higher quantities of NA to evoke any effects on intracellular Ca^2+^. As expected, the first response of NIH3T3 cells to NA was not an elevation but a reduction in intracellular [Ca^2+^]. The [Ca^2+^] increase following [Ca^2+^] reduction may be disrupted by ruining Ca^2+^ storage in the ER by TG, an endoplasmic reticulum Ca^2+^-ATPase pump inhibitor. Therefore, it was suggested that the overall effect of [Ca^2+^] increase may be divided into two steps. Firstly, NA reduces intracellular [Ca^2+^], possibly via triggering the efflux of Ca^2+^ ions out of the cell membrane channels. Secondly, the Ca^2+^ release from the ER contributes at least in part to the [Ca^2+^] elevation. Since small amounts of NA (1 mM) do not elevate but only reduce [Ca^2+^], the release of Ca^2+^ from the ER requires higher concentrations of NA to trigger this process.

The present study sought to elucidate the mechanisms underlying NA-induced changes in intracellular [Ca^2+^] waves. Based on other studies and the data of the present study, a hypothesis was proposed that the metabolism of NA adenine dinucleotide phosphate (NAADP) is important during NA modulation of cellular Ca^2+^. A putative synthesis pathway for NAADP exists, NAADP is a well established Ca^2+^ mobilizing agent that releases Ca^2+^ from intracellular stores (19). In the presence of NA and nicotinamide adenine dinucleotide phosphate (NADP), ADP-ribosyl cyclase catalyzes the synthesis of NAADP by a base exchange reaction and cAMP is a stimulator during this process (20–22). Notably, NA reduces the intracellular cAMP concentration (5). At a high concentration, NA may inhibit cAMP and thereby limit the synthesis of NAADP. The decrease of NAADP may be responsible for the first [Ca^2+^] drop upon exposure to NA. Excessive NA may rapidly overcome the cAMP-limited step and promote the synthesis of NAADP. Therefore, a marked [Ca^2+^] elevation was observed. Furthermore, a markedly high concentration of NA (100 mM) may completely eradicate cAMP and re-establish cAMP as a rate-limiting step. Therefore, the [Ca^2+^] elevation curve following 100 mM NA treatment is not as steep as that following 10 mM NA treatment. In the present study, 2 mM 8-Br-cAMP (a cAMP analog) delayed and alleviated the first [Ca^2+^] drop in response to NA, suggesting that cAMP has a key role in the changes in the [Ca^2+^] wave induced by NA.

It is well established that the intracellular Ca^2+^ wave may modulate the cytoskeletal structure (23–28). Although F-actins and microtubules underwent disassembly upon incubation with NA, the external addition of high concentrations of CaCl_2_ only disrupted the F-actin filaments. It was hypothesized that besides the [Ca^2+^] elevation, other pathways must also be involved in the disassembly of microtubules. The disruption of F-actin and microtubule cytoskeleton may definitely negatively effect the intracellular traffic process. In cultured cells, an opaque material accumulated around the nucleus when incubated with 70 mM NA. It appears that the minus end (nuclear region) to plus end (cell membrane region)-directed transport process was inhibited and therefore, cargo was deposited in the perinuclear region. Further evidence in the xenopus melanocyte system confirmed that NA induced an intracytic transport deficiency.

In conclusion, the present study showed that NA regulated the intracellular calcium concentration depending on its initial concentration and exposure time. High concentrations of nicotinic acid induced cytoskeletal disassembly and promoted β-tubulin degradation in a proteasome-dependent manner. The cytoskeletal disassembly may finally contribute to the disruption of the intracellular transport process. Further investigations aim to minimize the functional concentration of NA and characterize the function of NA in different biological systems, particularly in cancer cells and animal models. As the cytoskeleton is essential during cell migration and EMT, interrupting the dynamic arrangement of the cytoskeletion may break the fundamental cancerous processes of metastasis. NA provides potential for clinical use in the future.

## References

[1] Altschul R, Hoffer A, Stephen JD (1955). Influence of nicotinic acid on serum cholesterol in man. Arch Biochem Biophys.

[2] Carlson LA (2005). Nicotinic acid: the broad-spectrum lipid drug. A 50th anniversary review. J Intern Med.

[3] Figge HL, Figge J, Souney PF (1988). Nicotinic acid: a review of its clinical use in the treatment of lipid disorders. J Pharm Pharmacol.

[4] Offermanns S (2006). The nicotinic acid receptor GPR109A (HM74A or PUMA-G) as a new therapeutic target. Trends Pharmacol Sci.

[5] Gille A, Bodor ET, Ahmed K (2008). Nicotinic acid: pharmacological effects and mechanisms of action. Annu Rev Pharmacol Toxicol.

[6] Morrow JD, Parsons WG, Roberts LJ (1989). Release of markedly increased quantities of prostaglandin D2 in vivo in humans following the administration of nicotinic acid. Prostaglandins.

[7] Andersson RGG, Aberg G, Brattsand R (1977). Studies on the mechanism of flush induced by nicotinic acid. Acta Pharmacol Toxicol (Copenh).

[8] Benyó Z, Gille A, Kero J (2005). GPR109A (PUMA-G/HM74A) mediates nicotinic acid–induced flushing. J Clin Invest.

[9] Tunaru S, Kero J, Schaub A (2003). PUMA-G and HM74 are receptors for nicotinic acid and mediate its anti-lipolytic effect. Nat Med.

[10] Kostylina G, Simon D, Fey MF (2008). Neutrophil apoptosis mediated by nicotinic acid receptors (GPR109A). Cell Death Differ.

[11] Zhao S, Jiang H, Wang W (2007). Cloning and developmental expression of the Xenopus Nkx6 genes. Dev Genes Evol.

[12] Shi Y, Zhao S, Li J (2009). Islet-1 is required for ventral neuron survival in Xenopus. Biochem Biophys Res Commun.

[13] Downey GP, Chan CK, Trudel S (1990). Actin assembly in electropermeabilized neutrophils: role of intracellular calcium. J Cell Biol.

[14] Yoneda M, Nishizaki T, Tasaka K (2000). Changes in actin network during calcium-induced exocytosis in permeabilized GH3 cells: calcium directly regulates F-actin disassembly. J Endocrinol.

[15] Forscher P (1989). Calcium and polyphosphoinositide control of cytoskeletal dynamics. Trends Neurosci.

[16] Rosado JA, Sage SO (2000). The actin cytoskeleton in store-mediated calcium entry. J Physiol.

[17] Wilson MT, Kisaalita WS, Keith CH (2000). Glutamate-induced changes in the pattern of hippocampal dendrite outgrowth: a role for calcium-dependent pathways and the microtubule cytoskeleton. J Neurobiol.

[18] Sheets L, Ransom DG, Mellgren EM (2007). Zebrafish melanophilin facilitates melanosome dispersion by regulating dynein. Curr Biol.

[19] Yamasaki M, Churchill GC, Galione A (2005). Calcium signalling by nicotinic acid adenine dinucleotide phosphate (NAADP). FEBS J.

[20] Aarhus R, Graeff RM, Dickey DM (1995). ADP-ribosyl cyclase and CD38 catalyze the synthesis of a calcium-mobilizing metabolite from NADP. J Biol Chem.

[21] Wilson H, Galione A (1998). Differential regulation of nicotinic acid–adenine dinucleotide phosphate and cADP-ribose production by cAMP and cGMP. Biochem J.

[22] Rah SY, Mushtaq M, Nam TS (2010). Generation of cyclic ADP-ribose and nicotinic acid adenine dinucleotide phosphate by CD38 for Ca2+ signaling in interleukin-8-treated lymphokine-activated killer cells. J Biol Chem.

[23] Sobue K, Kanda K, Adachi J (1983). Calmodulin-binding proteins that interact with actin filaments in a Ca^2+^-dependent flip-flop manner: survey in brain and secretory tissues. Proc Natl Acad Sci USA.

[24] Shin DM, Zhao XS, Zeng W (2000). The mammalian Sec6/8 complex interacts with Ca^2+^ signaling complexes and regulates their activity. J Cell Biol.

[25] Constantin B, Meerschaert K, Vandekerckhove J (1998). Disruption of the actin cytoskeleton of mammalian cells by the capping complex actin-fragmin is inhibited by actin phosphorylation and regulated by Ca^2+^ ions. J Cell Sci.

[26] Brown SS, Yamamoto K, Spudich JA (1982). A 40,000-dalton protein from Dictyostelium discoideum affects assembly properties of actin in a Ca^2+^-dependent manner. J Cell Biol.

[27] Yamamoto H, Fukunaga K, Tanaka E (1983). Ca^2+^- and calmodulin-dependent phosphorylation of microtubule-associated protein 2 and tau factor, and inhibition of microtubule assembly. J Neurochem.

[28] Gradin HM, Marklund U, Larsson N (1997). Regulation of microtubule dynamics by Ca^2+^/calmodulin-dependent kinase IV/Gr-dependent phosphorylation of oncoprotein 18. Mol Cell Biol.

